# Multiple organ dysfunction syndrome in pediatric colchicine poisoning: a national cohort study from Türkiye

**DOI:** 10.55730/1300-0144.6204

**Published:** 2026-02-04

**Authors:** Faruk EKİNCİ, Dinçer YILDIZDAŞ, Özden ÖZGÜR HOROZ, Hayri Levent YILMAZ, Sevcan BİLEN, İlker ÜNAL, İkbal TÜRKER, Esra ŞEVKETOĞLU, Nihal AKÇAY, Agop ÇITAK, Güntülü ŞIK, Tanıl KENDİRLİ, Merve HAVAN, Murat KANGIN, Mehmet Nur TALAY, Nazik YENER, Hatice ALBAYRAK, Edin BOTAN, Utku ÖZER, Arzu OTO, Gürkan BOZAN, Muhterem DUYU, Başak Nur AKYILDIZ, Naime GÖKAY, Elif ÇELİK, Çapan KONCA, Mutlu Uysal YAZICI, Oğuz DURSUN, Feyza İNCEKÖY GİRGİN, Hatice Feray ARI, İlknur ARSLAN, Murat ÖZKALE, Özlem SANDAL, Merve MISIRLIOĞLU, Halil KESKİN, Sadık KAYA, Arda KILINÇ, Ferhat SARI, Fatih AKIN, Ayşe AŞIK, İbrahim BİNGÖL, Eda TURANLI, Merve BOYRAZ, Ener Çağrı DİNLEYİCİ, Mehmet Akif DÜNDAR, Selver Ceren ÖZCAN, Aygül KAYA AKILLI, Emine AKKUZU, Nazan ÜLGEN TEKEREK, Nilüfer ÖZTÜRK, Emrah GÜN, Yasemin ÖZKALE, Nurettin Onur KUTLU, Zeynelabidin ÖZTÜRK

**Affiliations:** 1 Division of Pediatric Intensive Care Unit, Department of Pediatrics, Faculty of Medicine, Çukurova University, Adana, Turkiye; 2 Division of Pediatric Emergency Care, Department of Pediatrics, Faculty of Medicine, Çukurova University, Adana, Turkiye; 3 Department of Medical Biostatistics, Faculty of Medicine, Çukurova University, Adana, Turkiye; 4 Division of Pediatric Intensive Care Unit, Department of Pediatrics, Bakırköy Dr. Sadi Konuk Training and Research Hospital, İstanbul, Turkiye; 5 Division of Pediatric Intensive Care Unit, Department of Pediatrics, Faculty of Medicine, Acıbadem University, İstanbul, Turkiye; 6 Division of Pediatric Intensive Care Unit, Department of Pediatrics, Faculty of Medicine, Ankara University, Ankara, Turkiye; 7 Division of Pediatric Intensive Care Unit, Department of Pediatrics, Gazi Yaşargil Training and Research Hospital, Diyarbakır, Turkiye; 8 Division of Pediatric Intensive Care Unit, Department of Pediatrics, Faculty of Medicine, Samsun 19 Mayıs University, Samsun, Turkiye; 9 Division of Pediatric Intensive Care Unit, Department of Pediatrics, Van Training and Research Hospital, Van, Turkiye; 10 Division of Pediatric Intensive Care Unit, Department of Pediatrics, Faculty of Medicine, The University of Health Sciences Bursa Yüksek İhtisas Training and Research Hospital, Bursa, Turkiye; 11 Division of Pediatric Intensive Care Unit, Department of Pediatrics, Faculty of Medicine, Eskişehir Osmangazi University, Eskişehir, Turkiye; 12 Division of Pediatric Intensive Care Unit, Department of Pediatrics, İstanbul Göztepe Prof. Dr. Süleyman Yalçın City Hospital, İstanbul, Turkiye; 13 Division of Pediatric Intensive Care Unit, Department of Pediatrics, Faculty of Medicine, Erciyes University, Kayseri, Turkiye; 14 Division of Pediatric Intensive Care Unit, Department of Pediatrics, Seyhan State Hospital, Adana, Turkiye; 15 Division of Pediatric Intensive Care Unit, Department of Pediatrics, Faculty of Medicine, Adnan Menderes University, Aydın, Turkiye; 16 Division of Pediatric Intensive Care Unit, Department of Pediatrics, Faculty of Medicine, Adıyaman University, Adıyaman, Turkiye; 17 Division of Pediatric Intensive Care Unit, Department of Pediatrics, Faculty of Medicine, Gazi University, Ankara, Turkiye; 18 Division of Pediatric Intensive Care Unit, Department of Pediatrics, Dr. Sami Ulus Obstetrics and Gynecology, Children’s Health and Disease Training and Research Hospital, Ankara, Turkiye; 19 Division of Pediatric Intensive Care Unit, Department of Pediatrics, Faculty of Medicine, Akdeniz University, Antalya, Turkiye; 20 Division of Pediatric Intensive Care Unit, Department of Pediatrics, Faculty of Medicine, Marmara University, İstanbul, Turkiye; 21 Division of Pediatric Intensive Care Unit, Department of Pediatrics, Şanlıurfa Training and Research Hospital, Şanlıurfa, Turkiye; 22 Division of Pediatric Intensive Care Unit, Department of Pediatrics, Adana City Training and Research Hospital, Adana, Turkiye; 23 Division of Pediatric Intensive Care Unit, Department of Pediatrics, Faculty of Medicine, Baskent University Seyhan Training and Research Hospital, Adana, Turkiye; 24 Division of Pediatric Intensive Care Unit, Department of Pediatrics, Faculty of Medicine, Health Sciences University Dr. Behçet Uz Children’s Disease and Surgery Training and Research Hospital, İzmir, Turkiye; 25 Division of Pediatric Intensive Care Unit, Department of Pediatrics, Samsun Training and Research Hospital, Samsun, Turkiye; 26 Division of Pediatric Intensive Care Unit, Department of Pediatrics, Faculty of Medicine, Atatürk University, Erzurum, Turkiye; 27 Division of Pediatric Intensive Care Unit, Department of Pediatrics, Hatay Training and Research Hospital, Hatay, Turkiye; 28 Division of Pediatric Intensive Care Unit, Department of Pediatrics, Başakşehir Çam and Sakura City Hospital, İstanbul, Turkiye; 29 Division of Pediatric Intensive Care Unit, Department of Pediatrics, Faculty of Medicine, İstanbul Aydın University, İstanbul, Turkiye; 30 Division of Pediatric Intensive Care Unit, Department of Pediatrics, Faculty of Medicine, Necmettin Erbakan University, Konya, Turkiye

**Keywords:** Colchicine, intensive care unit, organ dysfunction, pediatric, poisoning

## Abstract

**Background/aim:**

Colchicine poisoning has been associated with systemic toxicity, multiple organ dysfunction syndrome (MODS), and mortality. In the present study, the demographic, clinical, and laboratory characteristics of pediatric colchicine poisoning cases and the incidence of organ failure are investigated to identify potential risk factors associated with MODS.

**Materials and methods:**

Included in this retrospective multicenter cohort study were pediatric patients admitted to 28 tertiary-care pediatric intensive care units with a diagnosis of colchicine poisoning. Demographic data, clinical and laboratory findings, and clinical outcomes were extracted from medical records. MODS was defined as dysfunction involving at least two organ systems. Multivariable logistic regression analysis was performed to identify the variables independently associated with MODS.

**Results:**

Of the 150 patients included in the study, 40% were younger than 5 years and 52% were older than 13 years. Gastrointestinal symptoms were most prominent, with nausea and vomiting observed in 64.7%, abdominal pain in 36.7%, and diarrhea in 24%. Overall, 66% developed no organ failure, while single-organ failure occurred in 7.3% and 26.7% developed MODS. The coagulation system was most frequently affected (26%), followed by the hematological (19.3%) and immunological (18%) systems. Multivariable logistic regression analysis identified the baseline PRISM III score, ingested colchicine dose per kilogram of body weight, and initial sodium, creatinine, and aspartate aminotransferase (AST) levels as independent predictors of MODS.

**Conclusion:**

MODS can develop at ingestion doses lower than 0.5 mg/kg, which have historically been considered safe. Higher ingested doses and elevated PRISM III scores were associated with MODS, although these findings are derived from retrospective observational data and should not be interpreted as causal or as direct clinical recommendations. Further prospective studies are needed to clarify relevant doses, interventions, and prognostic factors in pediatric colchicine poisoning associated with MODS.

## 1. Introduction

Colchicine is a natural lipophilic alkaloid component extracted from the autumn crocus (*Colchicum autumnale*) that has for centuries been used for the management of acute gouty arthritis. Its mechanism of action involves the inhibition of microtubule polymerization, subsequently downregulating microtubule-based inflammatory processes, including chemotaxis. The antiinflammatory and antimitotic effects of colchicine in autoinflammatory diseases have led to its expanded use in many rheumatological conditions, such as Familial Mediterranean Fever (FMF), Behçet’s disease, scleroderma, psoriasis, sarcoidosis, amyloidosis, recurrent pericarditis, Sweet Syndrome, and certain spondyloarthropathies [1,2]. Although colchicine is generally safe if administered according to the established guidelines and recommended doses, it has a narrow therapeutic index, as the associated increased toxic risk limits its clinical use. Even chronic use at therapeutic daily doses may lead to adverse reactions in up to 20% of patients, including abdominal pain, diarrhea, nausea, and vomiting. However, these symptoms are generally mild, transient, and reversible after dose reduction [3]. Excessive dosing is associated with high rates of systemic toxicity, multiorgan failure, and even mortality, making colchicine poisoning one of the most serious clinical toxicologic emergencies [4–6]. Colchicine poisoning has most frequently been reported in the Mediterranean region and the Middle East due to the high prevalence of FMF and the resulting greater colchicine prescription rates. Nevertheless, most existing literature takes the form of case reports, case series, and small sample size studies, leaving significant gaps in our understanding of the clinical course, optimal treatment strategies, and outcomes, particularly within the pediatric population.

This multicenter study describes the demographic, clinical, laboratory, and treatment characteristics of pediatric colchicine poisoning cases admitted to pediatric intensive care units (PICU) in a Mediterranean country. The primary objective of this study was to determine the incidence of specific organ failures and to identify any factors associated with the development of multiple organ dysfunction syndrome (MODS) in a large pediatric study cohort.

## 2. Materials and methods

### 2.1. Study design

Data for this retrospective, multicenter observational cohort study were obtained from 28 PICUs in 15 university hospitals and 13 local training and research hospitals in Türkiye.

### 2.2. Inclusion criteria

Included in the study were children aged 1 month to 18 years with colchicine poisoning who were admitted to the PICUs of the participating centers between January 1, 2015 and December 31, 2022. The maximum recommended daily dose of colchicine is 2 mg, and any ingestion exceeding this can be considered indicative of overdose [7]. Accordingly, included in the study were cases with recorded single-dose ingestions greater than 2 mg and those with unknown ingestion amounts accompanied by severe clinical symptoms. Patients with concomitant overdoses of cardiotoxic, neurotoxic, or hepatotoxic medications or other substances (including antipsychotics, antiepileptics, and antihypertensive agents) were excluded from the study.

### 2.3. Data acquisition

A total of 150 patients who were treated in 28 centers were included in the study. Data were extracted both from the electronic medical records and traditional archives of the participating centers. The extracted data included age, sex, weight, ingestion dose (mg/kg, based on parent or patient report), concomitant drug intake, indication for colchicine use, presence of chronic disease, time from ingestion to hospital admission, duration of PICU and hospital stay, PICU mortality prediction scores (Pediatric Index of Mortality 3 [PIM 3] and Pediatric Risk of Mortality 3 [PRISM III]), clinical symptoms, initial laboratory parameters (complete blood count, electrolytes, liver and renal function tests, coagulation profile, blood gas analysis, and creatine kinase), and treatment modalities, including invasive procedures and pharmacological interventions.

Cases that developed organ failure during their PICU stay were also recorded, defined according to the Pediatric Organ Dysfunction Information Update Mandate (PODIUM) organ dysfunction criteria published in 2022 [8]. The PODIUM criteria relate to 10 organ systems: neurologic, cardiovascular, respiratory, gastrointestinal, acute liver, renal, hematological, coagulation, endocrine, and immune systems. The number of dysfunctional organs, organ dysfunction durations, and other organ failure assessment tools such as the Pediatric Sequential Organ Failure Assessment Score and Pediatric Logistic Organ Dysfunction score 2 were also noted [9,10]. The development of dysfunction in two or more organs at any time during the PICU stay was defined as new-onset MODS [11–13]. The study cohort was divided into two groups: patients with dysfunction of two or more organ systems were classified as MODS (+), whereas those with single-organ dysfunction or no organ dysfunction were classified as MODS (−).

### 2.4. Study approval

The study protocol was granted approval by the Medical Research Local Ethics Committee of the primary study center (Approval number: 125.8, September 16, 2022). All the participant centers obtained local institutional study approval for their involvement.

### 2.5. Statistical analysis

IBM SPSS Statistics (Version 26.0, IBM Corp., Armonk, NY, USA) was used for the statistical analysis. The distribution of numerical variables was investigated using visual and analytical methods to determine normality. Normally distributed variables were presented using mean ± standard deviation (SD), while median and quartiles were used for nonnormally distributed and ordinal variables. Independent samples t-tests or Mann–Whitney U tests were used for between-group comparisons, depending on the distribution. Descriptive data relating to categorical variables were presented as frequencies and percentages. The chi-square or Fisher’s exact test was used for the comparison of unordered categorical data, and a Mann–Whitney U test was used for the comparison of ordered (rank) data between the groups. Variables with a p-value of <0.15 were included in the multivariable logistic regression model for the identification of independent factors related to the occurrence of MODS in colchicine poisoning. A receiver operating characteristic (ROC) curve was drawn to define the optimal cut-off value for sensitivity and specificity in continuous variables with significant associations with the presence of MODS in colchicine poisoning. A p-value of less than 0.05 was considered statistically significant (two-tailed).

## 3. Results

### 3.1. Cohort characteristics

The demographic and clinical characteristics of the study population (n = 150) are summarized in Table 1. Females represented 74% of the cohort (111/150), while males accounted for 26% (39/150). Sixty patients (40%) were younger than 5 years of age whereas 78 patients (52%) were older than 13 years, and the median age was 13.6 years (interquartile range (IQR): 3.1–15.9 years). The median ingested colchicine dose was 0.23 mg/kg (IQR: 0.13–0.39 mg/kg). The ingested colchicine dose was <0.5 mg/kg in 128 patients (85.3%), 0.5–0.8 mg/kg in 18 patients (12%), and >0.8 mg/kg in four patients (2.7%).

Of the 150 patients, 82 (54.7%) had ingested colchicine in attempted suicide, while ingestion was unintentional in 68 (45.3%). The median age of the patients who ingested colchicine in a suicide attempt was 15.6 years (IQR: 14.5–16.5 years), compared with 2.9 years (IQR: 2.1–4 years) in those whose ingestion was unintentional (p < 0.001). The median time from drug intake to hospital admission was 3 h, ranging from 1 to 80 h. Only six patients (4%) were admitted to hospital more than 24 h after drug ingestion.

In the study cohort, 18 patients (12%) had multidrug exposure, with the most common coingested drugs being paracetamol and nonsteroidal antiinflammatory drugs, followed by antibiotics and selective serotonin reuptake inhibitors (SSRIs).

### 3.2. Signs & symptoms in the first 24 h

Among the study population, 108 patients (72%) developed at least one sign or symptom in the first 24 h of colchicine exposure. The most common manifestations were gastrointestinal symptoms, including nausea and vomiting (64.7%), followed by abdominal pain (36.7%) and diarrhea (24%) (Table 2). Isolated gastrointestinal symptoms were observed in 60 out of the 108 patients (56%) who developed symptoms within the first 24 h, while the remaining 48 patients (44%) exhibited gastrointestinal symptoms in combination with other systemic manifestations.

Patients who developed additional symptoms alongside gastrointestinal manifestations had ingested higher colchicine doses than those with isolated gastrointestinal symptoms (0.33 vs 0.29 mg/kg, p > 0.05), but the difference was not statistically significant. The rate of subsequent MODS development was 60.4% in the group that developed multiple gastrointestinal symptoms compared with 16.7% in patients with isolated manifestations.

### 3.3. Treatment

All patients received at least one treatment modality, and the most common interventions were activated charcoal (94.7%) and gastric lavage (88%). The treatments administered to the study population are presented in Table S1.

### 3.4. Organ failure

**Figure 1:**
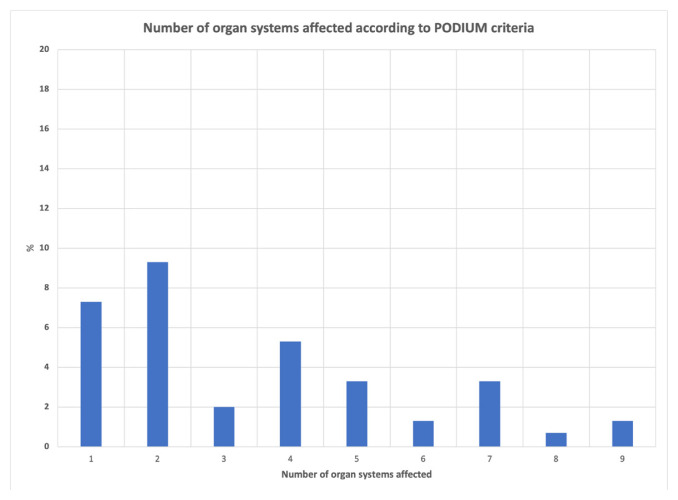
Number of organ systems affected according to the PODIUM criteria.

**Figure 2:**
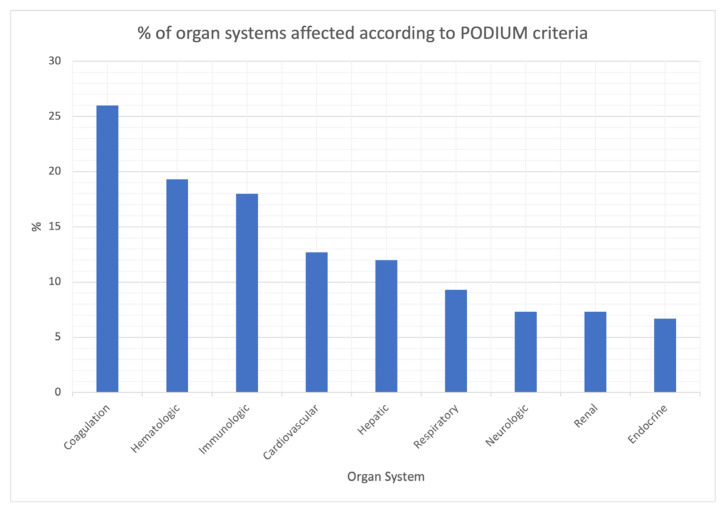
Percentage of organ systems affected according to the PODIUM criteria.

Overall, no organ failure was observed in 66% of the study population, while single-organ failure occurred in 11 (7.3%) and MODS in 40 (26.7%) (Figure 1). The coagulation system was most frequently affected (n = 39, 26%), followed by the hematological (n = 29, 19.3%) and immunological (n = 27, 18%) systems, and the cardiovascular (12.7%), hepatic (12%), respiratory (9.3%), neurologic (7.3%), renal (7.3%), and endocrine (6.7%) systems to a lesser degree (Figure 2).

The median ingested colchicine dose (mg/kg) was highest in the neurological and hematological involvement groups (0.41 mg/kg, and 0.40 mg/kg, respectively) followed by the cardiovascular involvement group (0.39 mg/kg), whereas the lowest doses were observed in the endocrine, coagulation, renal, and immunological involvement groups. The relationship between median ingested colchicine dose and organ system involvement is presented in Table 3.

### 3.5. Development of MODS

MODS developed in 40 patients (26.7%). The demographic, clinical, and initial laboratory parameters of the patients with and without MODS are listed in Table 1 and Table S2. The median age was 5.1 years in the MODS (−) group and 15.5 years in the MODS (+) group with a statistically significant difference (p < 0.001). The presence of any symptoms in the first 24 h was observed in 97.5% of patients in the MODS (+) group compared with 62.7% in the MODS (−) group (p < 0.001). The median colchicine dose per body weight was 0.22 mg/kg (IQR: 0.12–0.36 mg/kg) in the MODS (−) group and 0.27 mg/kg (IQR: 0.14–0.45 mg/kg) in the MODS (+) group (p=0.052). PIM 3 and PRISM III scores at PICU admission were also significantly higher in the MODS (+) group (Table 1).

**Figure 3:**
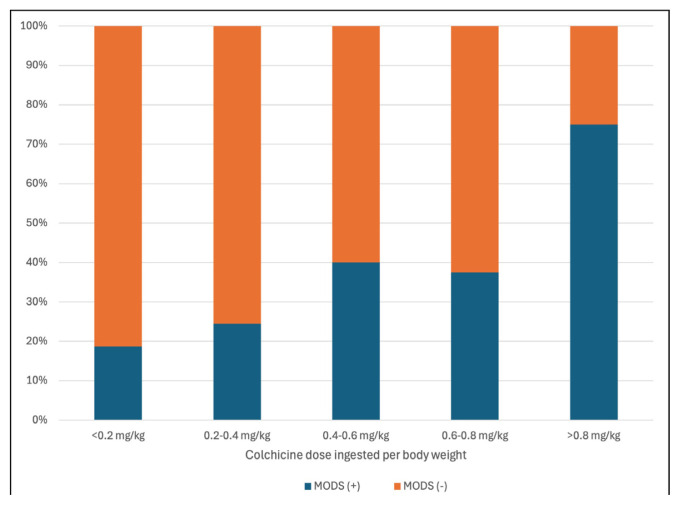
Percentage distribution of patients with MODS (+) and MODS (−), based on ingested colchicine dose/body weight (mg/kg). The figure shows the proportion of MODS status within each dose category.

Figure 3 illustrates the proportion of MODS status within each dose category.

Table S2 presents initial laboratory findings comparing patients with and without MODS. When the appropriate parameters in Table 1 and Table S2 were included in multivariable logistic regression analysis; initial PRISM III score, colchicine dose ingested per body weight, initial sodium, creatinine, and AST levels were found to be independently associated with MODS development (Table 4). Each 0.1 mg/dl increase in the initial creatinine level was found to be independently associated with 2.78 times increased risk (1.70–4.53) for MODS. The AUROC values to predict MODS development for colchicine dose (per kg) and PRISM III score were 0.60 (95% CI: 0.50–0.71), and 0.854 (95% CI: 0.78–0.92), respectively (Table S3). A colchicine dose cut-off of ≥0.19 mg/kg demonstrated 73% sensitivity and 47% specificity for predicting MODS in acute colchicine poisoning.

### 3.6. Outcomes

Most patients recovered without any sequelae (n = 142, 94.7%) whereas mortality was observed in five (3.3%). Long-term sequelae, including alopecia and mild muscle weakness, were observed in three patients (2%).

## 4. Discussion

Recalcitrant and progressive MODS is the most common antecedent for death in many PICUs. MODS occurs in 6–71% of critically ill children in PICUs, with day-1 prevalences typically ranging from 14% to 37%, depending on the applied criteria [14]. Although sepsis is reported to be the most common etiology of MODS, accounting for up to 45% of MODS cases in some cohorts, several other risk factors have also been included in the MODS etiology, such as severe hypoxemia, cardiorespiratory arrest, shock, trauma, acute pancreatitis, hematological malignancies, hematopoietic cell transplantation, envenomation, and toxic injury and poisoning [15–17]. Pediatric trauma is also recognized as a common reason for MODS in pediatric patients, with a high ratio of MODS (23.1%) being reported in a large pediatric trauma cohort on the day of PICU admission [18]. Specific rates for MODS in pediatric poisoning cases admitted to PICUs are not broadly documented in epidemiological studies, as the available data are mostly limited to case reports or small cohorts describing severe but variable organ involvement, including the liver and kidneys, and coagulopathy [19].

Overdoses of colchicine—a neutral and lipophilic alkaloid characterized by a narrow therapeutic index and potent antimitotic activity—impair cell division in the affected tissues, resulting in multiorgan involvement with a poor prognosis [20]. It is toxic to all tubulin-containing cells in the body, and mainly affects proliferative tissues such as the intestinal mucosa and hematopoietic system [21]. Myocardial injuries occur as a result of disruptions to the conduction and contraction of myocardial cells, whereas respiratory failures are generally caused by neuromuscular block [22].

The present study represents the largest pediatric colchicine poisoning cohort to date and is the first to specifically evaluate factors associated with the development of MODS. Our findings reveal initial PRISM III score, ingested colchicine dose (per weight), and laboratory parameters such as initial sodium, creatinine, and AST levels, to be independently associated with the development of MODS during PICU stay.

The first evidence of colchicine poisoning is seen during the first 24 h, with gastrointestinal symptoms such as vomiting, diarrhea, and abdominal pain. In our study, 108 patients (72%) developed symptoms within the first 24 h following exposure, with nausea/vomiting, abdominal pain, and diarrhea being by far the most common symptoms (64.7%, 36.7%, and 24%, respectively). Suzen-Orhan et al. reported that 16 (72,7%) of the 22 patients in their study became symptomatic after drug exposure, and that vomiting was the most common symptom, occurring in 13 (59%) patients, followed by diarrhea, abdominal pain, and fatigue [23]. Lower initial symptom rates have also been reported. In one study, only seven of 17 patients (41%) developed symptoms upon presentation to the hospital [24]. This lower rate of symptoms was possibly attributable to the early admission of patients, which was a mean 7.3 h after drug exposure, and the lack of data on any symptoms that developed following the initial presentation. In the present study, the first 24 h was not solely characterized by gastrointestinal symptoms, as other signs and symptoms (such as palpitation, tachycardia, hypotension, fatigue, alteration in consciousness, myalgia, fever, and respiratory distress) were also present in a large proportion of the symptomatic patients. A detailed analysis revealed that patients presenting with multiple symptoms had ingested higher doses of colchicine than those with only gastrointestinal symptoms. Furthermore, 60.4% of the participants in this group developed MODS, compared to 16.7% in the group that developed only gastrointestinal symptoms. This suggests that the presence of early nongastrointestinal symptoms may indicate more severe intoxication and a greater risk of subsequent organ failure. We believe that further studies should focus on the symptomatology of this potentially fatal drug exposure given the potential of early symptoms to predict subsequent developments of MODS during PICU stays, and to guide the selection and timing of advanced treatment modalities prior to the onset of MODS.

The distribution of ingested colchicine doses in the present study was as follows: 128 patients (85.3%) ingested <0.5 mg/kg, 18 (12%) ingested 0.5–0.8 mg/kg, and four (2.7%) ingested >0.8 mg/kg. The proportions of those that developed MODS during their PICU stay in these three groups were 25%, 27.8%, and 75%, respectively. It is thus apparent that the likelihood of MODS occurrence increases with the ingested dose, especially in ingestions over 0.8 mg/kg. A similar relationship between ingested dose and mortality has been reported in an earlier study [25].

Bismuth et al. reported minor toxicity with ingestions of less than 0.5 mg/kg, major toxicity with myelosuppression with 10% mortality following the ingestion of 0.5–0.8 mg/kg, and 100% mortality following ingestions over 0.8 mg/kg [4]. It would seem, however, that these cut-off ranges do not apply to pediatric patients. Ozdemir et al. reported three deaths (13%) in their 23 pediatric colchicine poisoning cohort, with doses of 0.5 mg/kg, 1.25 mg/kg, and 1.4 mg/kg [26]. Polat et al., in their study of pediatric patients, reported 100% mortality due to disseminated intravascular coagulopathy (DIC), shock, and multiorgan failure in three patients who had ingested doses of 0.5–0.84 mg/kg [27]. The above studies all suggest that pediatric mortality thresholds may be lower than those reported in adult studies, and the findings of the present study further support a reconsideration of these limits, given that MODS can develop even after low ingestion doses. In our cohort, the median colchicine dose was 0.27 mg/kg (IQR:0.14–0.45) in the MODS (+) group. Moreover, MODS occurred in 25% of the patients that ingested <0.5 mg/kg of colchicine, suggesting that one-quarter of children who ingest <0.5 mg/kg are at risk of MODS during PICU stay. Multivariate logistic regression analysis showed that every 0.1 mg/kg increase in the ingested colchicine dose increases the risk of MODS by 1.6 times (IQR: 1.17–2.22) in pediatric colchicine poisoning cases.

The patterns and frequencies of organ failure in pediatric colchicine poisoning have yet to be clarified in the literature. The most commonly affected organ system in adult studies differs from report to report. In one adult study, hematological involvement with blood marrow depression between days 3 and 6 was reported in 20 (27%) of 73 patients [4]. In a further study of 43 adult colchicine poisoning patients, acute respiratory distress syndrome (ARDS) was reported in 35%, renal failure in 35%, and cardiovascular involvement in 18.6% of the study population [5]. The frequency and patterns of organ failure remain uncertain as pediatric studies have generally included a relatively lower number of patients. In the present study, coagulation, hematological, and immunological system failures were the most common, followed by cardiovascular, hepatic, and respiratory involvement. Although gastrointestinal system (GIS) symptoms are generally the first manifestations in colchicine poisoning, no severe GIS failure was observed in our study cohort. This may be attributable to the absence of clearly defined clinical and laboratory findings during diagnosis, which may increase the risk of nonrecognition in critically ill patients.

There are several limitations to the present study, one of which is the lack of blood colchicine level data, and the study’s use of the maximum possible ingested dose identified based on the patient’s history. The retrospective design of the study, the heterogeneity of the study cohort based on dose ingested per weight, and the different treatment regimens applied by the many institutions may limit the generalizability of the findings. This variability in institutional treatment protocols and clinical management strategies may have also influenced patient outcomes, and could not be fully standardized or controlled for the study. Although ingested colchicine dose and PRISM III scores were associated with MODS development, these findings are based on observational data and should not be interpreted as causal relationships or direct clinical recommendations due to the potential effects of unmeasured confounders. Finally, the factors associated with mortality could not be analyzed due to the low mortality rate in the study group, and delayed sequelae, such as neurologic, respiratory, and cardiac complications, could not be assessed due to the lack of postdischarge follow-up.

## 5. Conclusion

The present study reveals an association between colchicine ingestion below 0.5 mg/kg and the development of MODS, although its findings are based on observational data and should not be treated as definite clinical recommendations. Even lower ingestion doses may warrant closer monitoring and supportive care within standard clinical practice. Although the PRISM III score was originally developed for the prediction of PICU mortality, our results support its use as a scoring system for the prediction of MODS in cases of pediatric colchicine poisoning; nevertheless, this observation does not establish clinical predictive utility. Further prospective studies are needed to clarify relevant doses, interventions, and prognostic factors in pediatric colchicine poisoning-associated MODS and mortality.

## Supplementary materials

**Table S1:** The treatments applied to the study cohort.

Treatment	n	%

Activated charcoal	142	94.7
• Multiple doses of activated charcoal	80	53.3

Gastric lavage	132	88

Antibiotics	36	24

Blood products transfusion	19	12.7

Vasopressors	16	10.7

Therapeutic plasma exchange	11	7.3

Continuous renal replacement therapy	11	7.3

Invasive mechanical ventilation	8	5.3

Granulocyte-colony stimulating factor	7	4.7

Noninvasive mechanical ventilation	5	3.3

Antiarrhythmic drugs	2	1.3

Extracorporeal membrane oxygenation	2	1.3

**Table S2:** Initial laboratory findings of patients with acute colchicine poisoning, comparing patients with multiple organ failure vs. no multiple organ failure.

Parameters	MODS (−) (n=110)	MODS (+) (n=40)	p-value
Hb (g/dL) *	12.5 ± 1.5	13 ± 2.9	0.288
**WBC (K/μL)**	8.8 (6.8–10.6)	10.3 (6.3–17.7)	0.061
**ANC (K/μL)**	4.5 (3.4–6.3)	7.3 (3.5–14.5)	**0.006**
**ALC (K/μL)**	2.7 (1.7–4.4)	2.2 (1.2–3.9)	0.113
**PLT (K/μL)**	304 (241–370)	230 (155–317)	**<0.001**
**PT (s)**	12.7 (12–14.1)	16.9 (13.9–21)	**<0.001**
**APTT (s)**	28 (25.1–32.8)	29.2 (24.8–39.2)	0.217
**Fibrinogen (mg/dL)**	248 (210–330)	232 (180–311)	0.152
**BUN (mg/dL)**	11 (9–14.2)	13.4 (10–24)	**0.016**
**Creatinine (mg/dL)**	0.45 (0.31–0.54)	0.65 (0.51–0.82)	**<0.001**
Uric acid (mg/dL) *	5.1 ± 1.9	3.7 ± 1.3	**<0.001**
**AST (U/L)**	31 (24–44)	65 (28–201)	**<0.001**
**ALT (U/L)**	17 (14–25)	24 (14–73)	0.052
**Total bilirubin (mg/dL)**	0.4 (0.2–0.6)	0.4 (0.3–0.7)	0.255
Na (mEq/L) *	139.3 ± 2.9	138.1 ± 4.1	**0.049**
K (mEq/L) *	4.1 ± 0.4	3.9 ± 0.7	0.090
Albumin (g/dL) *	4.2 ± 0.5	4.2 ± 0.7	0.952
**CPK (U/L)**	116 (76–167)	209 (112–538)	**<0.001**
**Troponin (ng/mL)**	0.004 (0.002–0.04)	0.13 (0.005–1.3)	**<0.001**
**CRP (mg/dL)**	0.3 (0.1–0.8)	0.8 (0.3–9.2)	**<0.001**
**pH**	7.38 (7.36–7.41)	7.36 (7.29–7.40)	**0.033**
**Bicarbonate (mEq/L)**	22 (20.6–23.6)	19.9 (18–22)	**<0.001**
**Lactate (mmol/L)**	1.33 (0.91–1.90)	2.15 (1.2–3.55)	**<0.001**

ALC, absolute lymphocyte count; ALT, alanine aminotransferase; ANC, absolute neutrophil count; APTT, activated partial thromboplastin time; AST, aspartate aminotransferase; BUN, blood urea nitrogen; CPK, creatine phosphokinase; Hb, hemoglobin; PCT, procalcitonin; PLT, platelets; PT, prothrombin time; WBC, white blood cell. Bold p-values indicate statistically significant differences between the MODS (−) and MODS (+) groups (p < 0.05).

**Table S3:** AUROC of colchicine dose ingested and initial PRISM 3 score according to presence of MODS.

Test result variable(s)	Area	Std. error	Asymptotic sig.	Asymptotic 95% confidence interval	
				Lower bound	Upper bound
Colchicine dose (mg/kg)	0.604	0.053	0.052	0.501	0.707
PRISM 3 score	0.854	0.036	0.000	0.784	0.924

## Figures and Tables

**Table 1:** General characteristics and clinical data of patients with acute colchicine poisoning by MODS status.

Variable	Total (n=150)	MODS (−) (n=110)	MODS (+) (n=40)	p-value

**Male, n (%)**	39 (26)	33 (30)	6 (15)	0.064

**Female, n (%)**	111 (74)	77 (70)	34 (85)	

**Age (years), median (IQR)**	13.6 (3.1–15.9)	5.1 (2.5–15.4)	15.5 (14–16.5)	**<0.001**

**Weight (kg), median (IQR)**	40 (14–55.3)	20 (13–50)	53.5 (45–60)	**<0.001**

**Unintentional ingestion, n (%)**	68 (45.3)	62 (56.4)	6 (15)	**<0.001**
**Suicide attempt, n (%)**	82 (54.7)	48 (43.6)	34 (85)	

**Time to admission (h), median (IQR)**	3 (1.4–6)	3 (1–5)	5 (3–9.75)	**<0.001**

**Any symptoms at first 24 h, n (%)**	108 (72)	69 (62.7)	39 (97.5)	**<0.001**

**Nongastrointestinal symptoms within the first 24 h, n (%)**	48 (32%)	19 (17.2)	29 (72.5)	**<0.001**

**Multidrug exposure, n (%)**	18 (12)	13 (11.8)	5 (12.5)	1.000

**Own medication, n (%)**	67 (44.4)	39 (35.5)	28 (70)	**<0.001**

**Total colchicine dose (mg), median (IQR)**	5 (3–13.8)	4.5 (2.5–10)	13 (7.1–25)	**<0.001**

**Colchicine dose (mg/kg), median (IQR)**	0.23 (0.13–0.39)	0.22 (0.12–0.36)	0.27 (0.14–0.45)	0.052

**Classification per dose, n (%)**				
	
• **<0.5 mg/kg**	128 (85.3)	96 (75)	32 (25)	0.083
• **0.5–0.8 mg/kg**	18 (12)	13 (72.2)	5 (27.8)	
• **>0.8 mg/kg**	4 (2.7)	1 (25)	3 (75)	

**Hospital stay (days), median (IQR)**	5 (3–7)	4 (3–6)	8 (7–13)	**<0.001**

**PICU stay (days), median (IQR)**	3 (3–5)	3 (2–4)	6 (4–9.75)	**<0.001**

**PIM 3 score at admission, median (IQR)**	1.1 (0.4–1.5)	0.9 (0.3–1.4)	1.5 (1–3.8)	**<0.001**

**PRISM III score, median (IQR)**	3 (0–7)	2 (0–4)	8.5 (4.5–10)	**<0.001**

**PELOD score, median (IQR)**	0 (0–2)	0 (0–1)	4.5 (2–11)	**<0.001**

**pSOFA, median (IQR)**	0 (0–3)	0 (0–1)	5 (4–8)	**<0.001**

**Number of organ failures according to PODIUM, median (IQR)**	0 (0–2)	0 (0–0)	4 (2–6)	**<0.001**

PELOD, Pediatric Logistic Organ Dysfunction; PICU, pediatric intensive care unit; PIM 3, Pediatric Index of Mortality-3; PRISM III, Pediatric Risk of Mortality-3; pSOFA, Pediatric Sequential Organ Failure Assessment Score; PODIUM, Pediatric Organ Dysfunction Information Update Mandate. Bold p-values indicate statistically significant differences between the MODS (−) and MODS (+) groups (p < 0.05).

**Table 2:** Signs & symptoms and clinical findings of the study population.

Symptom	n	%	Clinical findings	n	%
Nausea and vomiting	97	64.7	Metabolic acidosis	20	13.3
Abdominal pain	55	36.7	Rhabdomyolysis	17	11.3
Diarrhea	36	24	Sepsis	8	5.3
Palpitation and/or tachycardia	32	21.3	Arrhythmia	7	4.7
Hypotension	15	10	Acute pancreatitis	4	2.6
Fatigue	13	8.7			
Alteration in consciousness	12	8			
Myalgia	10	6.7			
Fever	9	6			
Respiratory distress	5	3.3			
Other	6	4			

**Table 3:** Development of organ failures according to median colchicine dose (mg/kg) ingested.

Organ failure	n (%)	Colchicine dose ingested per weight (mg/kg) median (IQR)
**Neurologic**	11 (7.3)	0.41 (0.23–0.48)
**Hematological**	29 (19.3)	0.40 (0.21–0.53)
**Cardiovascular**	19 (12.7)	0.39 (0.19–0.56)
**Respiratory**	14 (9.3)	0.35 (0.12–0.50)
**Liver**	18 (12)	0.35 (0.13–0.45)
**Immune system**	27 (18)	0.30 (0.17–0.50)
**Renal**	11 (7.3)	0.30 (0.19–0.44)
**Coagulation**	39 (26)	0.30 (0.13–0.48)
**Endocrine**	10 (6.7)	0.30 (0.13–0.45)

**Table 4:** Multivariable logistic regression analysis of factors affecting progression to MODS.

Variables	OR (95% CI)	p-value
**PRISM III score**	1.55 (1.26–1.91)	<0.001
**Colchicine dose per kg (per 0.1 mg/kg)**	1.61 (1.17–2.22)	0.004
**Sodium (per 1 mEq/L decrease)**	1.36 (1.07–1.72)	0.012
**Creatinine (per 0.1 mg/dL)**	2.78 (1.70–4.53)	<0.001
**AST (per 10 U/L)**	1.28 (1.01–1.61)	0.039
